# Underreporting of Energy Intake Increases over Pregnancy: An Intensive Longitudinal Study of Women with Overweight and Obesity

**DOI:** 10.3390/nu14112326

**Published:** 2022-06-01

**Authors:** Katherine M. McNitt, Emily E. Hohman, Daniel E. Rivera, Penghong Guo, Abigail M. Pauley, Alison D. Gernand, Danielle Symons Downs, Jennifer S. Savage

**Affiliations:** 1 Center for Childhood Obesity Research, The Pennsylvania State University, University Park, PA 16802, USA; katherinemcnitt@gmail.com; 2 Control Systems Engineering Laboratory, Arizona State University, Tempe, AZ 85281, USA; daniel.rivera@asu.edu (D.E.R.); penghong.guo114@gmail.com (P.G.); 3 Exercise Psychology Laboratory, The Pennsylvania State University, University Park, PA 16802, USA; amp34@psu.edu (A.M.P.); dsd11@psu.edu (D.S.D.); 4 Department of Nutritional Sciences, The Pennsylvania State University, University Park, PA 16802, USA; adg14@psu.edu; 5 Center for Childhood Obesity Research, Department of Nutritional Sciences, The Pennsylvania State University, State College, PA 16801, USA; jfs195@psu.edu

**Keywords:** obesity, gestational weight gain, prenatal care, eating behaviors, stress, mHealth

## Abstract

(1) Background: Energy intake (EI) underreporting is a widespread problem of great relevance to public health, yet is poorly described among pregnant women. This study aimed to describe and predict error in self-reported EI across pregnancy among women with overweight or obesity. (2) Methods: Participants were from the Healthy Mom Zone study, an adaptive intervention to regulate gestational weight gain (GWG) tested in a feasibility RCT and followed women (*n* = 21) with body mass index (BMI) ≥25 from 8–12 weeks to ~36 weeks gestation. Mobile health technology was used to measure daily weight (Wi-Fi Smart Scale), physical activity (activity monitor), and self-reported EI (MyFitnessPal App). Estimated EI was back-calculated daily from measured weight and physical activity data. Associations between underreporting and gestational age, demographics, pre-pregnancy BMI, GWG, perceived stress, and eating behaviors were tested. (3) Results: On average, women were 30.7 years old and primiparous (62%); reporting error was −38% ± 26 (range: −134% (underreporting) to 97% (overreporting)), representing an ~1134 kcal daily underestimation of EI (1404 observations). Estimated (back-calculated), but not self-reported, EI increased across gestation (*p* < 0.0001). Higher pre-pregnancy BMI (*p* = 0.01) and weekly GWG (*p* = 0.0007) was associated with greater underreporting. Underreporting was lower when participants reported higher stress (*p* = 0.02) and emotional eating (*p* < 0.0001) compared with their own average. (4) Conclusions: These findings suggest systemic underreporting in pregnant women with elevated BMI using a popular mobile app to monitor diet. Advances in technology that allow estimation of EI from weight and physical activity data may provide more accurate dietary self-monitoring during pregnancy.

## 1. Introduction

Two-thirds of women enter pregnancy with overweight or obesity [1], and over 60% will exceed gestational weight gain (GWG) recommendations [2]. Women who enter pregnancy with elevated BMI and/or exceed GWG recommendations are at risk for complications including gestational diabetes, preeclampsia, unsuccessful breastfeeding, and postpartum weight retention [3,4,5,6], and longer-term risks such as type 2 diabetes and some cancers [7,8]. In offspring, risks include macrosomia, large for gestational age, high blood pressure, and obesity [9,10,11]. Additionally, many people do not consume key nutrients during pregnancy and improved dietary guidance is warranted to help pregnant people to meet but not exceed dietary recommendations [12].

The Institute of Medicine recommends clinical dietary assessment for all pregnant people [13] and this may be especially beneficial for those at risk of excessive GWG [14]. Clinicians ask patients to monitor their food and energy intake (EI) [13,15]. In the general population, underreporting of EI is widespread [16,17] and is positively associated with BMI, younger age, and psychosocial factors, including cognitive restraint [18,19,20,21]. However, studies of underreporting during pregnancy are lacking. Underreporting of EI makes it difficult for health care providers to accurately interpret and monitor self-reported dietary information and may result in ineffective intervention efforts to regulate GWG.

Estimated prevalence of underreporting during pregnancy ranges from 13% to 50%, with the highest prevalence among those with pre-pregnancy overweight and obesity [22,23,24]. These studies relied on cross-sectional data and used a variety of methods to estimate underreporting (e.g., threshold cutoffs) to exclude “implausible” reporters [25], which collapses quantifiable underreporting (e.g., kcal, percent EI) into categorical groups (e.g., over reporters, under-reporters, “adequate” reporters) based on arbitrary limit values. Threshold cutoffs and cross-sectional data limit our understanding of how EI changes across trimesters in pregnancy as nutritional needs change. In sum, prior research focused primarily on identifying inadequate reporters in cross-sectional studies while the estimated magnitude of dietary underreporting during pregnancy remains unknown.

This study’s aim was to describe the extent of energy intake reporting error throughout pregnancy among women with overweight or obesity using an intensive longitudinal data approach [26]. We also examined maternal factors associated with underreporting (i.e., demographics, pre-pregnancy BMI, GWG, perceived stress, and eating behaviors). Based on previous literature in pregnant and non-pregnant samples, we hypothesized underreporting would be positively associated with gestational age [27], income [27,28], pre-pregnancy BMI, GWG [27,28], perceived stress [29], uncontrolled eating [29], and emotional eating [29]. We also expected underreporting to be negatively associated with maternal age [27,28] and dietary restraint [27,28,29].

## 2. Materials and Methods

### 2.1. Study Subjects

Participants were pregnant women in the Healthy Mom Zone study, an adaptive intervention to regulate GWG tested in a feasibility randomized control trial and followed pregnant women with overweight and obesity (*n* = 21) from early pregnancy to ~36 weeks gestation living in and around State College, PA (ClinicalTrials.gov identifier #NCT03945266) [30]. This was an optimization trial within the multiphase optimization strategy (MOST) framework [31]. Details of the Healthy Mom Zone study intervention have been published previously [32]. Participants were recruited from 2016–2017 through flyers, online platforms, and referrals by local obstetricians at first prenatal appointment. Inclusion criteria were 8–12 weeks gestation and pre-pregnancy BMI = 24.5–45.0 (BMI = 40–45 were enrolled with physician consent). Exclusion criteria included pre-existing diabetes and other conditions known to impact fetal growth or GWG, severe allergies or dietary restrictions, contraindications to prenatal physical activity, and not residing in the area. Thirty-one participants were randomized to either the intervention (*n* = 15) or standard of care control (*n* = 16). All participants (*n* = 31) received usual prenatal health care through their personal health care provider and the intervention offered nutrition and physical activity guidance beyond what was offered in standard care. Regardless of group randomization, participants completed study measures daily, weekly, and monthly throughout the study. From this initial group, one participant was missing all EI data, one dropped out, one was non-compliant (e.g., <70% of measures completed), three had a first trimester miscarriage, and four had BMI < 25.0, resulting in a final sample size of 21 for this analysis. Ethical approval for the Healthy Mom Zone study was granted by the Pennsylvania State University Institutional Review Board (STUDY00003752, approval date: 12/1/15), participants provided written informed consent to participate, and all aspects of data collection and storage were in accordance with standards stipulated by this body. 

### 2.2. Measures

#### 2.2.1. Demographic Characteristics

At baseline, demographics and self-reported pre-pregnancy weight were collected from participants using questionnaires and trained nurses obtained height. Gestational age was defined using the first day of last menstrual cycle.

#### 2.2.2. Weight and Physical Activity Measures

Participants weighed themselves daily from home using a Fitbit Aria Wi-Fi Smart Scale (Fitbit Inc., San Francisco, CA, USA). Weekly weight change was calculated as the average weekly weight minus the average weight of the prior week. Final maternal weights within 10 days of delivery were abstracted from medical records or using Aria Wi-Fi Smart Scale data if medical record data were not available. Total GWG was calculated for participants with a final maternal weight (*n* = 19) by subtracting self-reported pre-pregnancy weight from last available weight (within 10 days of delivery).

#### 2.2.3. Psychosocial Measures

At study enrollment and every four weeks thereafter, participants completed the 21-item Eating Inventory [33] via online surveys collected with the secure data platform, Research Electronic Database Capture (REDCap) [34]. The Eating Inventory, which has a four point response scale ranging from (1) definitely true to (4) definitely false, measures three eating behavior subscales: cognitive restraint (e.g., “I consciously hold back on how much I eat at meals to keep from gaining weight.”), uncontrolled eating (e.g., “Sometimes when I start eating, I just can’t seem to stop.”), and emotional eating (e.g., “I start to eat when I feel anxious.”). Scores for each subscale were calculated by averaging items. Internal consistencies ranged from acceptable to excellent (restrained eating: α = 0.71, uncontrolled eating: α = 0.86, emotional eating = 0.92). Participants completed the 10-item Perceived Stress Scale [35] at enrollment and weekly thereafter. The Perceived Stress Scale assesses how unpredictable, uncontrollable, and overloaded respondents find their lives (α = 0.89).

#### 2.2.4. Self-Reported Energy Intake

Self-reported EI was obtained using MyFitnessPal (dietary intake application). While MyFitnessPal is not a validated method for collecting EI, it was chosen due to its ease of use and acceptability among participants as a tool for self-monitoring [36]. Both intervention and control participants were trained on using the app and recorded all foods and drinks consumed over 24 h on three days per week (two weekdays and one weekend day). Resting metabolic rate (RMR) was estimated daily using quadratic formula: RMR = 0.1976(weight in kg)^2^ – 13.424(weight in kg) + 1457.6 [37]. This formula accounts for an assumed increase in RMR across gestation [37,38]. Physical activity (e.g., daily activity time, daily step count, and estimated energy expenditure) was assessed at baseline and throughout the study using a wrist-worn actigraphy device (Jawbone UP 4, Jawbone Inc., San Francisco, CA, USA) [39]. Jawbone UP 4 has been found to reliably predict physical activity, compared with other popular fitness monitors [40,41].

### 2.3. Calculating Underreporting of Energy Intake

In response to limited accuracy of self-reported EI, we expanded an energy balance model developed by Thomas and colleagues to back-calculate EI from GWG during pregnancy [28] using additional input variables, including measured daily weights (measured from home using Aria Wi-Fi Scale), activity kcal (Jawbone activity monitor), and resting metabolic rate (RMR) [38,42]. K_1_ and K_2_ are coefficients that map changes in daily energy intake and physical activity, respectively, into maternal weight gain/loss. T is the sampling time (in this case daily). The equation accounts for fetal and placental growth and expansion of the uterus, mammary glands, blood, and extracellular fluid in coefficients as a function of gestational age in days (*k*).

$$EI_{est}\left(k\right)=\frac{-W\left(k+2\right)+8W\left(k+1\right)-8W\left(k-1\right)+W\left(k-2\right)}{12TK_{1}}-\frac{K_{2}}{K_{1}}\left(PA\left(k\right)+RMR\left(k\right)\right)$$

To calculate reporting error, self-reported and back-calculated EI data were matched by date. Unmatched data were excluded from analyses. Reporting error was calculated using the equation: Reporting Error = [(self-reported EI-back-calculated EI)/back-calculated EI] × 100% [43]. This continuous variable represents error in reporting of EI or discrepancy between self-reported and back-calculated kcal. This includes participant error in reporting as well as potential inherent errors in the app database, and is reflective of what users experience when using a dietary tracking mobile app. Negative values indicate EI underreporting and positive values indicate over reporting, with 0 representing accurate reporting.

### 2.4. Statistical Analysis

Statistical analysis was performed in SAS version 9.4 (SAS Institute Inc., Cary, NC, USA). Sample means were calculated for continuous demographic variables (pre-pregnancy BMI, GWG, and age). Frequencies and percentages were calculated for categorical demographic variables (pre-pregnancy BMI category, race, ethnicity, marital status, employment status, income, gravidity, and parity). Survey data where participants reflected back on a prior period of time (e.g., Perceived Stress Scale) had study week assigned to the week prior to survey completion. Weekly and daily data were merged by gestational age and monthly and daily/weekly data were merged by study week. Restrained, emotional, and uncontrolled eating and perceived stress were mean-centered by participant to disaggregate the effect of within- and between-person fluctuations on reporting error.

Multilevel modeling [44] tested whether reporting error changed over time (i.e., gestational age) and associations with the following: anthropometrics (pre-pregnancy BMI, GWG), treatment group (intervention or control), demographics (maternal age, parity, household income), perceived stress, and eating behaviors (cognitive restraint, uncontrolled eating, and emotional overeating). Repeated observations (level 1) were nested within participant (level 2). Each model used restricted maximum likelihood, compound symmetry covariance structure (CS), and included gestational age was a covariate [45]. Linear, quadratic, and cubic effects of gestational week were considered. Post-hoc group comparisons were adjusted using Tukey method. Intraclass correlation coefficients (ICCs) were calculated as the ratio of between-subjects variance to total variance. Statistical significance was determined at *p* < 0.05.

## 3. Results

### 3.1. Demographic Data

Age at study entry ranged from 24–37 years (*M =* 30.7 ± 3.0). All subjects had overweight or obesity with a mean pre-pregnancy BMI = 32.7 ± 6.8. Forty-eight percent reported having overweight pre-pregnancy (BMI = 25.0–29.9 kg/m^2^) and 52% had obesity (BMI ≥ 30 kg/m^2^). Most participants were married (90%), primiparous (62%), well-educated (95% with a college degree or higher), affluent (76% reported an annual household income ≥ $40,000), and employed full-time (81%). Mean total GWG for this sample was 21.5 ± 15.4 kg (kg) (Intervention: *M =* 10.7 ± 7.0 kg, Control: *M =* 8.7 ± 7.3 kg) (Table 1).

### 3.2. Error in Reporting of Energy Intake

The mean of all reporting error observations (*n* = 1404) of −38% ± 26 (range: −134% (underreporting) to 97% (overreporting)), representing an approximately 1134 kcal underestimation daily. The ICC indicates about 54% of variation in reporting error variable was within-person, while 46% of variation was between-person. In other words, 54% of variance in reporting error is accounted for by change within participants (e.g., from day to day), while the remaining variation can be explained by characteristics differing between participants, such as pre-pregnancy BMI. Participant mean reporting error was −38% (range: −65–0%); meaning participants underreported EI by 38%. Twenty out of 21 participants underreported 90% of the time or more.

### 3.3. Change in Reporting Error across Pregnancy

**Figure 1:**
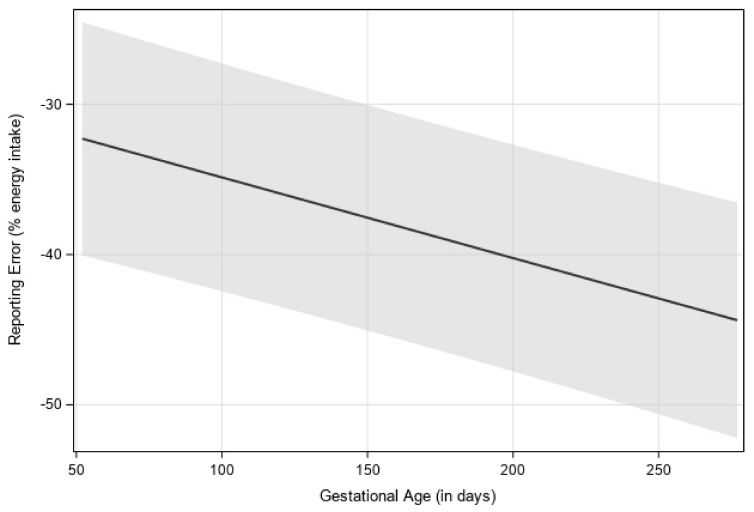
Visualization of estimated reporting error over gestational age (in days), with 95% confidence interval. Estimates were generated by using multilevel modeling (SAS PROC MIXED). Linear, quadratic, and cubic effects of gestational week were considered, with a linear relationship having the best model fit.

Mean self-reported EI did not significantly differ between first (*M =* 1792 ± 70), second (*M =* 1681 ± 67), and third trimesters (*M =* 1692 ± 68). Back-calculated EI increased by an average of 272 kcal from first (*M =* 2688 ± 144) to second trimester (*M =* 2960 ± 141; *p* < 0.0001) and 117 kcal from second to third trimester (*M =* 3077 ± 142; *p* = 0.0005) (Table 2). There was a between-person relationship between gestational age (in days), when treated as a continuous variable, on reporting error such that underreporting increased as pregnancy progressed (*p* < 0.0001) (Figure 1).

In a separate model, gestational age was examined as a categorical variable where there was a main effect of trimester on reporting error (*p* < 0.0001). Reporting error in the first trimester (LS mean = −32% ± 4) was significantly higher than in the second (−39% ± 4) and third trimesters (−40% ± 4).

### 3.4. Independent Factors Associated with Reporting Error

**Figure 2:**
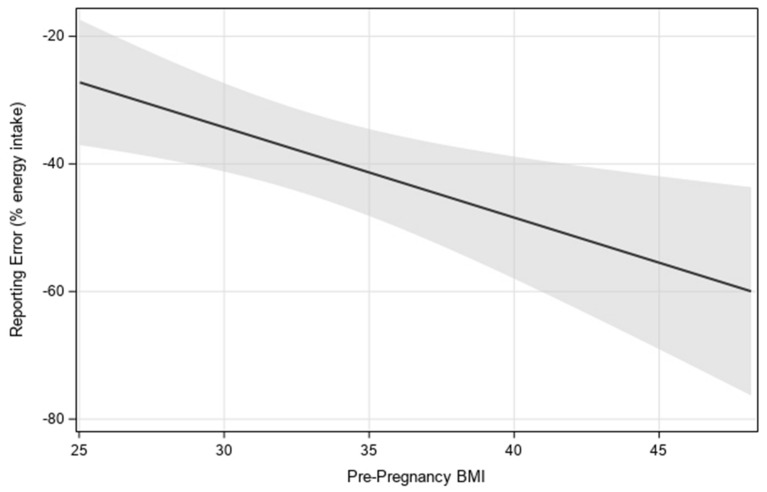
Visualization of the linear relationship between estimated reporting error and pre-pregnancy BMI, with 95% confidence interval. Estimates were generated by using multilevel modeling (SAS PROC MIXED).

A main effect of continuous pre-pregnancy BMI on reporting error showed higher pre-pregnancy BMI was associated with more underreporting (*p* = 0.01) (Figure 2). In a separate model, there was also a main effect of categorical pre-pregnancy BMI status on reporting error between participants with obesity (LS mean = −47% ± 4) and overweight (LS mean = −28% ± 5) (*p* = 0.0075). Mean self-reported EI did not significantly differ between participants with obesity (LS mean = 1637 ± 92) and overweight (LS mean = 1743 ± 97; *p* = 0.43), while mean back-calculated EI was lower in participants with overweight (LS mean = 2537 ± 165) compared with those with obesity (LS mean = 3324 ± 157; *p* = 0.0027).

While there was no association between overall GWG and underreporting, we observed a positive association between weekly GWG and underreporting (*p* = 0.0007), such that participants with higher weekly GWG had greater mean underreporting (Table 3). Additionally, when examining weekly GWG as a categorical variable, reporting error was lower in participants who exceeded (LS mean = −40.1% ± 4) compared with participants who were below (LS mean = −36% ± 4) weekly Institute of Medicine GWG recommendations based on trimester and BMI category (*p* = 0.0009) (Table 3). Three participants developed gestational diabetes mellitus (GDM) after enrollment in the trial. These women also had the highest pre-pregnancy BMIs of the sample. Sensitivity analyses were conducted excluding these participants (*n* = 18). All conclusions were the same, except that when participants with GDM were excluded, the positive association between total GWG and underreporting became statistically significant (*p* = 0.01).

Stress increased (*p* < 0.0001), while emotional, uncontrolled, and restrained eating decreased (all *p* < 0.05) across pregnancy. The ICC for perceived stress was 57%, indicating 43% of variability in stress was within- and 57% was between-person. After controlling for gestational age, a main effect of participant mean-centered perceived stress on reporting error showed that on days when participants reported higher stress compared with their own average, reporting error was more positive, indicating less underreporting (*p* = 0.02) (Table 3). The ICC for emotional eating was 81%, indicating 19% of variability in stress was within- and 81% was between-person. There was not a significant association between participants’ average emotional eating and average reporting error (*p* = 0.8). However, there was a significant effect of within-person emotional eating on reporting error, such that on days when participants reported higher emotional eating compared with their own average, underreporting was lower (*p* < 0.0001). ICCs for restrained and uncontrolled eating were 58% and 82%, respectively. Cognitive restraint and uncontrolled eating were not significantly associated with reporting error. While there was no significant relationship between treatment group and reporting error, there was an interaction of study group with weight status on reporting error (*p* = 0.01). Post hoc comparisons indicated that, in the intervention group, participants with overweight had lower underreporting than participants with obesity, suggesting that the intervention had a positive impact on underreporting for participants with overweight only. No significant relationships were detected between maternal age, parity, or income and underreporting (Table 3).

## 4. Discussion

This is the first study to use daily longitudinal data to characterize reporting accuracy in a sample of U.S. pregnant persons with elevated BMI, showing that underreporting increases throughout pregnancy. Further, pre-pregnancy BMI was positively associated with underreporting in the second trimester in this sample of women with overweight and obesity. Data also indicate that weekly GWG was positively associated with underreporting. Finally, higher than average perceived stress and emotional eating were associated with reporting error during pregnancy, but parity, age, income, cognitive restraint, and uncontrolled eating were not associated with reporting accuracy (Table 4). Together, these data suggest that underreporting has complex roots and the extent of underreporting increases later in pregnancy, despite simultaneous increases in recommended energy requirements to support fetal growth.

Across pregnancy, underreporting appeared to be driven by stable, self-reported EI. Back-calculated EI data indicate that participants consumed about 400 more kcal on average in trimester three, compared with trimester one, but self-reported eating the same amount of food across trimesters. This is consistent with a prior study showing EI underreporting prevalence was higher in late compared with early pregnancy [27]. People may tire of logging intake and reporting may become less accurate over time [46]. Dietary self-monitoring can be burdensome, resulting in non-compliance and underestimation [47], potentially explaining the increase in underreporting across pregnancy. Alternative methods of collecting dietary intake data, including remote food photography, are gaining popularity but further validation studies are needed [48].

This study adds to research showing underreporting is associated with pre-pregnancy BMI, with many of the previous studies on this topic including a majority of women with normal weight [27,49,50,51]. Though there was no significant relationship between total GWG and underreporting in this sample, we observed a positive relationship between changes in weekly GWG and underreporting. Higher weekly GWG may lead to increased underreporting through desirability bias. Meanwhile, underreporting could result in difficulty in self-monitoring and weight management. In contrast, Shiraishi found underreporters had lower total GWG when compared with normal- reporters [52]. More research is needed to elucidate the relationship between GWG and underreporting.

Psychological factors such as social desirability, eating restraint, and history of dieting are associated with underreporting in non-pregnant populations [29]. In addition, Moran found that limiting food intake to lose weight and self-reported dissatisfaction with weight/body shape were predictors of underreporting at 36-weeks’ gestation [27]. Very few studies have explored trends in restrained, emotional, and uncontrolled eating across pregnancy. One study found that dietary restraint was lower in the third trimester in comparison with the first, but no change in emotional eating [49].

Less is known about relationships between stress and underreporting during pregnancy, although positive associations were found in non-pregnant samples [29]. Contrary to our hypothesis, within-person fluctuations in perceived stress and emotional eating were negatively associated with underreporting in this sample. Emotionally salient information is typically better remembered than neutral information [53], and individuals with emotional eating have been shown to report greater dietary intake than individuals without emotional eating [54], especially during times of perceived stress [55]. This seems to be independent of dietary intake in non-pregnant samples [56]. For many people, pregnancy is a time of increased psychological distress [57]. Individual differences have been observed in food intake response to stress, with approximately 40% increasing, 40% decreasing, and 20% not changing dietary intake [58]. There may be something unique about prenatal stress that produces a tendency to reduce dietary intake, thus providing less opportunity for reporting error.

A variety of factors have been attributed to poor reporting of EI, including incomplete recordkeeping, conscious underreporting, changes in eating behavior from diet tracking, training, and quality control [29]. Common advice during pregnancy is to snack more often to meet additional kcal needs or combat morning sickness, and this may contribute to underreporting [59]. Future studies should explore additional factors that may influence within-person variation in underreporting which may include day of week (e.g., weekend vs. weekday), types of foods (e.g., snacks, beverages), selective underreporting of nutrients (e.g., fat or carbohydrates), frequency of consumption (e.g., unplanned eating, snacking), and other factors which vary from day to day.

MHealth technologies are increasingly popular among both healthcare providers and patients [60]. While the use of dietary and weight-tracking mobile apps, including MyFitnessPal, for self-monitoring of EI and weight have produced clinically significant weight loss in randomized controlled trials of non-pregnant people [61], our findings suggest users of diet-tracking apps may have difficulty self-monitoring intake due to systematic underreporting. Improving connectivity between weight, physical activity, and dietary mobile data would allow for use of predictive equations to back calculate EI within mHealth apps to give users a better understanding of their actual dietary intake and clinician guidance in counseling women during pregnancy to better manage weight.

In contrast to previous studies [27,28], we found no significant association of reporting error with the following: age, income, parity, or total GWG. Moran found socioeconomic status was an independent predictor at 36 weeks of EI underreporting. McGowan found young women were more likely to underreport than older women during pregnancy [51]. Thomas found higher income predicted higher underreporting [28]. One explanation for lack of association in our study is we had a relatively small, homogenous sample, which reduced our ability to detect relationships with demographics. Further research should explore characteristics associated with underreporting across gestation.

Findings from this study have important implications for behavioral interventions and research on dietary intake in pregnancy. Our data reinforce that underreporting is pervasive during pregnancy, especially in individuals with obesity. Participants in this sample underreported by an average of 986 kcal in trimester one, 1280 kcal in trimester two, and 1386 kcal in trimester three. Prenatal clinicians and intervention specialists should incorporate methods to improve reporting accuracy (e.g., multiple-pass 24 h recalls) [62] and be aware of social desirability bias in underreporting (e.g., higher BMI/gestational age). If self-reported EI is habitual, baseline self-reported EI may be an important indicator of participant consciousness level and sustained intervention efficacy. Finally, using predictive equations to estimate back-calculated EI may be a useful clinical and research tool, considering prevalence and magnitude of underreporting.

Strengths of this study include intensive longitudinal data collected throughout pregnancy, using reporting error as a continuous variable, as well as using measured weight and physical activity to determine back-calculated EI. There are also significant limitations to the results of this study. Limitations to this research include reliance on self-reported pre-pregnancy weight, which can lead to underestimated BMI [63]. In addition, the small sample size precludes the ability to make assumptions at a population level. Differences between actual and reported EI were calculated using an equation of approximation rather than gold standard measures (e.g., doubly labeled water). Although the equation accounts for factors relevant to weight change and gestational age in pregnancy, the equation relies on several assumptions (e.g., fetal physical activity in the womb is negligible) and does not account for all potential factors that can influence GWG (e.g., medications, genetics, obstetric complications). Finally, this was a homogenous sample of participants who were predominantly educated, non-Hispanic white, married, and middle-to-upper income, from central Pennsylvania, and enrolled in a GWG intervention, thus limiting the generalizability of the study findings to other populations of pregnant persons. Future research may extend these findings with a larger, more diverse sample. Research should also continue to explore interventions that promote reporting accuracy during pregnancy to improve patient adherence to EI recommendations to manage GWG.

## 5. Conclusions

Energy balance is essential for weight management during pregnancy, though this is difficult to monitor due to poor reporting of EI. Using a predictive equation to estimate EI, we found that underreporting using a popular diet-tracking mobile app was positively associated with pre-pregnancy BMI, weekly GWG, and gestational age across pregnancy, and negatively associated with perceived stress and emotional eating. These findings have implications for research and prenatal nutrition counseling and there is a need to develop efficacious interventions that improve reporting accuracy during pregnancy to promote maternal and child health. Research should also continue to explore which tools are most effective in improving reporting accuracy to promote positive pregnancy outcomes in individuals with overweight and obesity.

## Figures and Tables

**Table 1:** Baseline Descriptive Characteristics of Pregnant Women with Overweight and Obesity (*n* = 21).

Characteristic	N(%) ^1^
Maternal Age, years	30.7 ± 3.0
Preconception BMI, kg/m^2^	32.7 ± 6.8
% BMI = 24.5–29.9	10 (48%)
% BMI ≥ 30	11 (52%)
Gestational Age at Baseline (Weeks)	10.0 ± 1.7
Gestational Weight Gain, kg	21.5 ± 15.4
Race	
White	21 (100%)
Ethnicity	
Non-Hispanic	21 (100%)
Marital Status	
Divorced	1 (5%)
Married	19 (90%)
Single	1 (5%)
Maternal Education	
High School	1 (5%)
College	11 (52%)
Graduate/Professional School	9 (43%)
Gravidity	
1	11 (52%)
2	8 (38%)
3	2 (10%)
Parity	
0	13 (62%)
1	8 (38%)
Employment	
Full-Time	17 (81%)
Part-Time	2 (9%)
Self-Employed	1 (5%)
Other	1 (5%)
Household Income	
<$20,000	1 (5%)
$20,000–$40,000	4 (19%)
$40,000–100,000	8 (38%)
≥$100,000	8 (38%)

^1^ Continuous variables (maternal age and BMI: body mass index) data presented as mean plus/minus standard deviation.

**Table 2:** Energy Intake (kcal/d) and Underreporting During Pregnancy by Maternal Characteristics and Treatment Group in Pregnant Women with Overweight and Obesity.

Characteristic	Self-Reported EI (kcal/d) Mean ± SD	Back-Calculated EI, (kcal/d) Mean ± SD	Difference between Back-Calculated and Self-Reported EI, (kcal/d) Mean ± SD	% Underreporting Mean ± SD
Overall (*n* = 21)	1696 ± 481	2950 ± 142	1263 ± 162	38% ± 4
Gestational Age (Trimester)				
First Trimester	1702 ± 70 ^a^	2688 ± 144 ^a^	986 ± 166 ^a^	32% ± 4 ^a^
Second Trimester	1681 ± 67 ^a^	2960 ± 141 ^b^	1280 ± 162 ^b^	39% ± 4 ^b^
Third Trimester	1692 ± 68 ^a^	3077 ± 142 ^c^	1386 ± 164 ^c^	40% ± 4 ^b^
Pre-Pregnancy BMI				
BMI 25–29.9 (*n* = 10)	1743 ± 97 ^a^	2537 ± 165 ^a^	794 ± 190 ^a^	28% ± 5 ^a^
BMI ≥ 30 (*n* = 11)	1637 ± 92 ^a^	3324 ± 157 ^b^	1688 ± 181 ^b^	47% ± 4 ^b^
Total GWG Classified by Institute of Medicine Guidelines				
Not Exceeding (*n* = 12)	1736 ± 88 ^a^	3006 ± 191 ^a^	1271 ± 220 ^a^	35% ± 5 ^a^
Exceeding (*n* = 9)	1622 ± 102 ^a^	2874 ± 221 ^a^	1253 ± 254 ^a^	41% ± 6 ^a^
Parity				
0 (*n* = 13)	1672 ± 86 ^a^	3000 ± 184 ^a^	1329 ± 210 ^a^	40% ± 5 ^a^
1 (*n* = 8)	1712 ± 110 ^a^	2867 ± 234 ^a^	1156 ± 340 ^a^	36% ± 6 ^a^
Annual Household Income				
$10,000–$20,000 (*n* = 1)	1465 ± 315 ^a^	4286 ± 613 ^a^	2821 ± 61 ^a^	65% ± 17 ^a^
$20,000–$40,000 (*n* = 4)	1689 ± 158 ^a^	2695 ± 307 ^b^	1007 ± 346 ^b^	32% ± 9 ^a^
$40,000–$100,000 (*n* = 8)	1624 ± 111 ^a^	2971 ± 216 ^b^	1348 ± 244 ^b^	41% ± 6 ^a^
>$100,000 (*n* = 8)	1778 ± 111 ^a^	2888 ± 217 ^b^	1111 ± 244 ^b^	35% ± 6 ^a^
Treatment Group Assignment				
Intervention (*n* = 11)	1689 ± 94 ^a^	2902 ± 200 ^a^	1213 ± 229 ^a^	37% ± 5 ^a^
Control (*n* = 10)	1686 ± 99 ^a^	3002 ± 210 ^a^	1318 ± 240 ^a^	40% ± 6 ^a^

Values are least squared mean plus/minus standard error from repeated measures models (PROC MIXED). Results of statistical models are represented by a, b, c group comparisons. Values with different subscripts indicate a statistically significant difference between the two values (e.g., *p* < 0.05).

**Table 3:** Predictors of maternal underreporting of energy intake during pregnancy in women with overweight and obesity ^a^ (*n =* 25).

Variable	Model Estimate	Standard Error	*p*-Value
Gestational Age (days)	−0.05372	0.009664	<0.0001
Gestational Age (by trimester) (reference = Trimester 3)			<0.0001
Trimester (1)	8.0931	1.6027	
Trimester (2)	1.3743	1.1605	
Pre-Pregnancy BMI (kg/m^2^)	−1.4144	0.4943	0.0100
Pre-Pregnancy BMI classification (reference = BMI > 30)			0.0075
BMI = 25.0–29.9	19.3786	6.4793	
Perceived Stress (within-person)	0.2561	0.1033	0.0133
Perceived Stress (between-person)	−0.1708	0.6372	0.7915
Emotional Eating (within-person)	7.3520	0.5073	<0.0001
Emotional Eating (between-person)	−0.1734	0.6583	0.7950
Cognitive Restraint (within-person)	0.6897	0.5186	0.1838
Cognitive Restraint (between-person)	−2.7578	3.2976	0.4134
Uncontrolled Eating (within-person)	−0.3294	0.2798	0.2393
Uncontrolled Eating (between-person)	−1.3742	1.1126	0.2318
Total GWG (in kg) (*n =* 19)	−0.5049	0.5856	0.4006
Total GWG (meeting vs. exceeding Institute of Medicine guidelines) (reference = meeting guidelines)	−6.5364	8.3740	0.4458
Weekly GWG (in kg)	−5.4802	0.9972	<0.0001
Weekly GWG (meeting vs. exceeding Institute of Medicine guidelines) (reference = meeting guidelines)			0.0007
Under	5.0148	1.9283	
Over	0.5328	1.9144	
Treatment group (reference = intervention)			0.7294
Control	−2.7539	7.28448	
Maternal Age (yrs)	0.3989	1.3283	0.7672
Parity (reference = 1)			0.6131
Parity (0)	−4.1338	8.0404	
Household Income (yearly) (reference ≥ $100,000)			0.3396
$10,000–$20,000	−30.4517	18.3616	
$20,000–$40,000	2.8334	10.6118	
$40,000–100,000	6.9350	8.6440	

^a^ Multilevel model parameter estimates showing independent predictors of maternal reporting error, each in a separate model. All models controlled for gestational age except where gestational age/trimester was the predictor of interest.

**Table 4:** Summary of associations between participant characteristics and energy intake underreporting.

Predictor	Relationship with underreporting
Gestational age	Greater underreporting in later pregnancy
Pre-pregnancy BMI	Greater underreporting with higher BMI
Gestational weight gain	Greater underreporting with greater weekly weight gain
Maternal age	No association
Parity	No association
Household Income	No association
Perceived stress	Less underreporting during weeks when participant indicated higher stress than their usual stress level
Emotional eating	Less underreporting during months when participant indicated higher emotional eating than their usual level
Cognitive restraint	No association
Uncontrolled eating	No association

## Data Availability

Data will be made available upon request.
